# Detection and Phylogenetic Characterization of a Novel Herpesvirus in Sooty Terns *Onychoprion fuscatus*

**DOI:** 10.3389/fvets.2020.00567

**Published:** 2020-08-27

**Authors:** Manrico Sebastiano, Daniele Canestrelli, Roberta Bisconti, Anne Lavergne, Kévin Pineau, Olivier Chastel, Vincent Lacoste, David Costantini

**Affiliations:** ^1^Centre d'Etudes Biologiques de Chizé (CEBC), UMR 7372 CNRS-Univ, La Rochelle, France; ^2^Department of Ecological and Biological Science, Tuscia University, Viterbo, Italy; ^3^Laboratoire des Interactions Virus-Hôtes, Institut Pasteur de la Guyane, Cayenne, France; ^4^Groupe d'Etude et de Protection des Oiseaux en Guyane (GEPOG), Rémire-Montjoly, France; ^5^Unité de Biologie des Infections Virales Emergentes, Centre International de Recherche en Infectiologie, Institut Pasteur, Lyon, France; ^6^Unité Physiologie moléculaire et adaptation (PhyMA), Muséum National d'Histoire Naturelle, CNRS, CP32, Paris, France

**Keywords:** alphaherpesvirus, French Guiana, *Onychoprion fuscatus*, seabirds, disease

## Abstract

Since 2005, we have recorded annual episodes of alphaherpesvirus outbreaks in chicks of magnificent frigatebird *Fregata magnificens* on the Ile du Grand Connétable Nature Reserve in French Guiana. In 2009, we found sooty terns, *Onychoprion fuscatus*, that live sympatrically with frigatebirds, with visible clinical signs of a potential viral infection. To determine if the symptoms observed in sooty terns could be associated with an alphaherpesvirus previously identified in frigatebirds, we carried out molecular screening of samples collected from seven individuals. We identified and characterized a novel viral sequence from five birds. BLAST searches, pairwise nucleotide, and amino acid sequence comparisons, as well as phylogenetic analyses confirmed that the sequence belonged to the *Herpesviridae* family, of the *Alphaherpesvirinae* subfamily. We observed that it clustered with strains isolated from Podargidae (Caprimulgiformes), Columbiformes, and Falconiformes, but was distinct from the frigatebird herpesvirus. We have tentatively named it *Onychoprion fuscatus* alphaherpesvirus 1, (OfusAHV1). These two sequences, although found syntopic on the Ile du Grand Connétable, belong to two distinct alphaherpesvirus strains. Thus, the clinical symptoms showed by sooty terns do not likely result from a cross-species transmission event. Future work is needed to better characterize the virus and to investigate herpesvirus prevalence in healthy, free-ranging sooty terns, and to assess the impact of the virus on population viability.

## Introduction

Herpesviruses are DNA viruses found in many animal species, from invertebrates to mammals (1). Herpesviruses are thought to have evolved in association with their hosts. However, some studies reported cases of cross-species transmission, indicating that such events could occur more frequently than previously thought (2–4). These “spillover” infections in alternative hosts can result in dramatic outbreaks of disease (5–7). Because of their ability to establish a latent infection, herpesviruses do not generally pose a threat to their host species. However, some viruses can cause severe diseases and induce high mortality rates in their natural hosts (8, 9). This is the case for avian herpesviruses that remain one of the major causes of fatal infectious diseases in many bird species (10–12).

In 2005, we found several chicks of magnificent frigatebird *Fregata magnificens* on the Ile du Grand Connétable Nature Reserve (4°49' 36” N, 51°56' 38” W), a rocky island located off the coast of French Guiana, that showed clinical cutaneous signs or were found dead (13). In particular, chicks showed nodular proliferative skin lesions in legs and in the neck, and hyperkeratosis (13). A few years later, we characterized a novel alphaherpesvirus sequence from those chicks (13). In the following years, we have started a monitoring program of the population of frigatebirds, and have found that the disease is widespread in chicks, causing a number of physiological alterations associated with a high mortality rate (14–16). Since the first appearance of clinical signs in frigatebirds, we have also started annual monitoring programs for the other species that breed sympatrically in the natural reserve. On the 30th of April 2009, we found several dead or dying adult sooty terns *Onychoprion fuscatus* showing similar clinical signs of frigatebirds (bone frailty, hyperkeratosis) as described previously (13, 16, 17). Our goal was to determine if the observed symptoms could be due to a cross-species transmission of the alphaherpesvirus that affect magnificent frigatebirds or the results of an infection with an unknown herpesvirus.

## Materials and Methods

### Sample Collection

To determine if the observed symptoms could be due to a cross-species transmission we collected biological material (i.e., tracheal swabs and blood) from sick birds, while small tissue samples (i.e., trachea, brain, lungs, liver, and heart) from dead birds were additionally collected. Trachea (3 samples), brain (6 samples), lung (1 sample), liver (4 samples) heart (1 sample), and whole blood (4 samples) for a total of 19 samples were collected and placed in 2 mL Eppendorf tubes. Blood was centrifuged in the field and all samples were subsequently frozen in dry ice while in the field and were then kept in a −80°C freezer until laboratory analyses.

### Virus Identification

We extracted DNA by a classical phenol, phenol-chloroform (1:1 vol/vol), and chloroform technique and precipitated it by isopropanol. Then, we washed the DNA with 70% ethanol and resuspended it in TE buffer containing 10 mM Tris (pH 8.0) and 1 mM EDTA. We carried out molecular screening by semi-nested PCR amplifications with degenerate consensus primers targeting highly conserved amino acid motifs of the herpesvirus *DNA polymerase* gene. To this end, we used two sets of primers [First set: Freg1F: GTGTTCGATTTTGCCAGCCTGTATCC, Freg1R: ATGTTCCTTCCTATGGTCGTTACC, Freg2R: ACGTGCAGACACGGCAGAAG; Second set: as explained in (18)] targeting the same region of the gene, but with different levels of degeneracy. This was done for each DNA sample in separate reactions for the first-round PCR (Freg1F/Freg1R or DFASA/GDTD1B) and second-round PCR (Freg1F/Freg2R or VYGA/GDTD1B). The initial round of PCR contained 500 ng of genomic DNA, 30 pmoles of degenerate primers, 2 mM MgCl_2_, 0.2 mM each dNTP, 5 μL of 10 × PCR buffer, and 0.5 μL of AmpliTaq Gold DNA polymerase in a volume of 50 μL. We used 2 μL of this reaction in the semi-nested reaction. The PCR cycling conditions were as follows: after the DNAs were denaturated at 94°C for 10 min, the reaction mixtures were cycled five times at 94°C for 30 s, 60°C for 30 s, and 72°C for 30 s, followed by 30 cycles at 94°C for 30 s, 46°C for 30 s, and 72°C for 30 s. We made an extension of 10 min at 72°C on the last cycle (GeneAmp PCR system 9600 thermal cycler; Perkin-Elmer). Amplification products of the expected size (about 250 and 350 base pair, respectively) were cloned into pCR4-TOPO vectors using a TA cloning kit from Invitrogen and sent them for sequencing to Genewiz (https://www.genewiz.com/). For each PCR product, three clones of the “screening amplicons” were sequenced on both strands.

### Phylogenetic Analysis

Raw sequences were analyzed and edited in MEGA 5.05 (19). The nucleotide sequence was 307 bp in size, excluding primers. We then carried out sequence homology analyses using the BLAST program at the National Center of Biotechnology Information (NCBI) (http://blast.ncbi.nlm.nih.gov/Blast.cgi). Then, a multiple sequence alignment was constructed using ClustalW with previously published avian herpesvirus sequences and representative sequences for each genus or subfamily retrieved from GenBank (http://www.ncbi.nlm.nih.gov/nucleotide) (13, 20). The alignment was checked manually.

We analyzed the phylogenetic relationships among herpesviruses using the Bayesian inference (BI) approach implemented in Beast 1.8.4 (21), based on a final alignment including 37 unique sequences of 104 amino acid positions. We assessed the best-fit model of amino acid evolution for the dataset using the smart model selection (SMS) approach (22) as implemented in the PhyML environment (23). We ran BI analyses with LG+G+I model of aminoacidic substitution (24), an uncorrelated relaxed molecular clock model with a log-normal distribution (25), and a Yule tree prior. Then, we analyzed the results from two independent runs of 10 million generations, sampled every 1,000 generations, using Tracer 1.7.1 to check that the effective sample sizes for all parameters that exceeded 200 (26) and to assess the appropriate number of initial trees to discard as burn-in. Then, we combined the two runs using Logcombiner 1.8.4 [BEAST package, (27)]. We computed the Maximum Clade Credibility (MCC) tree summarizing the post-burn-in trees using TreeAnnotator 1.8.4, and we visualized the tree using FigTree 1.4.4 (26).

## Results and Discussion

**Figure 1:**
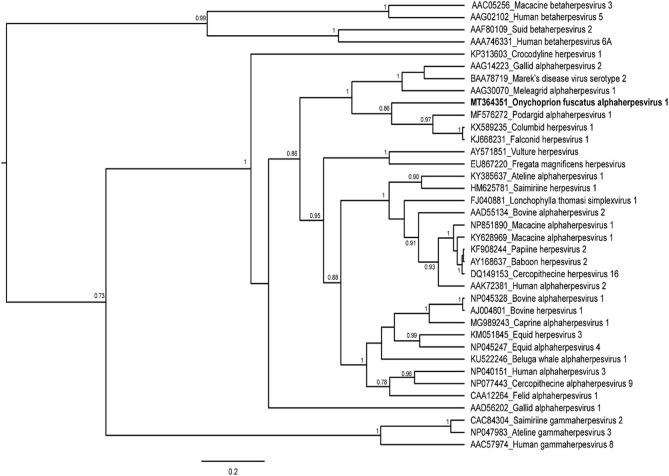
Bayesian inference of the phylogenetic relationships of the herpesvirus sequence identified in the sooty tern *Onychoprion fuscatus*. Bayesian posterior probabilities are shown at the nodes, when above 0.70. The sequence of *Onychoprion fuscatus* alphaherpesvirus 1 is in boldface. Alphanumerical codes represent the Genbank accession numbers of each herpesvirus sequence used in this study.

This study aimed at assessing the presence of a herpesvirus from dead and dying adult sooty terns and to determine its relationship with other members of the *Herpesviridae* family. Out of the 19 tissue samples, 7 samples (4 blood samples and 3 brain samples, collected from both dead and dying birds) tested positive for herpesvirus. A unique and novel viral sequence was obtained from five out of the seven individuals with PCR positive results. This sequence is tentatively designated as *Onychoprion fuscatus* alphaherpesvirus 1 (OfusAHV1) in the Alphaherpesvirinae subfamily. From a phylogenetic perspective, *Ofus*AHV1 clustered with strains detected from Podargidae (Caprimulgiformes), Columbiformes, and Falconiformes (posterior probability = 0.86; Figure 1). We also found that this novel alphaherpesvirus sequence of sooty terns was distinct from the Frigatebird herpesvirus (nucleotide p-distance: 0.28), which belonged to a different well-supported monophyletic lineage. These results seem to indicate that no cross-species transmission has occurred between frigatebirds and sooty terns. This is not surprising given that herpesviruses are usually associated with a single host species (28), and co-evolve with their host over long periods of time (28).

Because herpesviruses establish latent infections and have a high prevalence in natural hosts (11), symptoms of herpesvirus infection and the associated appearance of clinical signs may only occur when birds undergo a stressful situation (29). This raises the question of whether sooty terns were undergoing any form of stress and/or immune suppression. The severe clinical signs found in dying birds with positive PCR results may also suggest that sooty terns had no prior contact with this specific virus. Although very little is known about this population, recent work showed that sympatrically breeding frigatebirds have high blood concentrations of mercury while sooty terns showed very low mercury concentrations (30, 31). Mercury exposure can therefore likely be ruled out as a plausible candidate stressor for this population. However, suppression of the immune system in these birds might also be due to malnutrition, as has previously been suggested and recently corroborated in frigatebirds (13, 17).

This study does not conclusively prove the causal link between this herpesvirus and the occurrence of clinical signs and mortality in this population of sooty terns. The number of tissue samples and birds included in the present study was limited, and additional work would prove beneficial for a more solid interpretation of the results. However, the viral sequence here reported is novel and may be well-specific to this species, further supporting the fact that distinct avian populations are naturally infected with distinct herpesviruses. Seabirds aggregate at high densities during the breeding season, which may favor viral spread among conspecific and may lead to severe outbreaks in wild populations. We do not know the effect that this virus may have on this and other populations of sooty terns in terms of reproductive success and survival. However, given that herpesviruses in wild animals are usually only detected when they cause disease outbreaks, future works are needed to investigate herpesvirus prevalence in healthy, free-ranging seabirds. Seabirds are currently facing a strong decline in food resources (32, 33) and an increasing exposure to environmental contaminants and plastic pollution (34–36) which may increase their susceptibility to viral outbreaks.

## Data Availability Statement

The datasets presented in this study can be found in online repositories. The names of the repository/repositories and accession number(s) can be found at: https://www.ncbi.nlm.nih.gov/genbank/ (MT364351).

## Ethics Statement

The animal study was reviewed and approved by Prefet de la region Guyane.

## Author Contributions

MS wrote the article. DCa, RB, and DCo performed phylogenetic analyses. AL and VL performed virus identification analyses. KP and OC contributed to data collection. All authors contributed to the article and approved the submitted version.

## Conflict of Interest

The authors declare that the research was conducted in the absence of any commercial or financial relationships that could be construed as a potential conflict of interest.
